# Clinical factors influencing normalization of prothrombin time after stopping warfarin: a retrospective cohort study

**DOI:** 10.1186/1477-9560-6-15

**Published:** 2008-10-16

**Authors:** Sam Schulman, Rajae Elbazi, Michelle Zondag, Martin O'Donnell

**Affiliations:** 1 Department of Medicine, McMaster University, Hamilton, ON, Canada; 2 Faculty of Pharmaceutical Science, Utrecht University, Utrecht, The Netherlands

## Abstract

**Background:**

Anticoagulation with warfarin should be stopped 4–6 days before invasive procedures to avoid bleeding complications. Despite this routine, some patients still have high International Normalized Ratio (INR) values on the day of surgery and the procedure may be cancelled. We sought to identify easily available clinical characteristics that may influence the rate of normalization of prothrombin time when warfarin is stopped before surgery or invasive procedures.

**Methods:**

Clinical data were collected retrospectively from consecutive cases from two cohorts, who stopped warfarin 6 days before surgery. An INR value of 1.6 or higher on the day of surgery or requirement for reversal with vitamin K the day before surgery were criteria for slow return (S) to normal INR.

**Results:**

Of 202 patients, 14 (7%) were classified as S. Eight of the S-patients required reversal with vitamin K one day before surgery and in another case surgery was cancelled due to high INR. Baseline INR was the only variable significantly associated with classification as S in stepwise logistic regression analysis (p = 0.003). The odds ratio for being in the normal group was 0.27 (95% confidence interval 0.12–0.62) for each unit baseline INR increased. The positive predictive value of baseline INR with a cut off at > 3.0 was only 15% and for INR > 3.5 it was 33%.

**Conclusion:**

Baseline INR, but not the size of the maintenance dose, is associated with the rate of normalization of prothrombin time after stopping warfarin, but it has limited utility as predictor in clinical practice. Whenever normal hemostasis is considered crucial for the safety, the INR should be checked again before the invasive procedure.

## Background

With increasing life expectancy, and thereby also increasing prevalence of atrial fibrillation, a larger proportion of the population is using vitamin K antagonists (VKA). It has been reported that 36% of patients, 50 to 99 years of age, with atrial fibrillation are using VKA with a gradual increase over time [1]. Atrial fibrillation is prevalent in about 2.3 million people in the United States and one million are receiving warfarin [2]. In addition, other important groups of patients, such as those with venous thromboembolism or mechanical heart valve prosthesis use VKA.

Vitamin K antagonists are temporarily stopped to allow for invasive procedures. Therapeutically active anticoagulation with VKA during total knee arthroplasty (start of VKA 10 to 14 days preoperatively) was demonstrated to cause a greater need for blood transfusions and a lower postoperative hematocrit than a regimen without any effect of VKA (started the evening before surgery) during surgery [3]. In this context it is crucial to understand the rate of decline of the anticoagulant effect when VKA are discontinued. The normalization of the prothrombin time, expressed as International Normalized Ratio (INR) as an indicator of re-establishment of normal hemostasis depends on the clearance of the VKA but also on the capacity of the liver to synthesize the coagulation factors II, VII, IX and X. The latter is also contingent on the availability of vitamin K.

The clearance of VKA differs widely between the agents, from a half-life of 10 h for acenocoumarol, via 35 to 45 h for warfarin and up to 3 to 5 days for phenprocoumon. The most widely used VKA is warfarin, of which the most active S-enantiomer is hydroxylated by the hepatic microsomal enzyme CYP2C9 and the less active R-enantiomer is hydroxylated by CYP3A4, CYP1A1 and CYP1A2 [4]. Genetic polymorphisms in the CYP2C9 enzyme have been reported to affect the dose requirement of warfarin [4] but may not influence the normalization of prothrombin time after discontinuing warfarin. Instead, as discussed above, the capacity of the liver, availability of vitamin K and possibly other factors such as the sensitivity of the target enzyme VKOR*C1 *[5] are important for regeneration of functionally active coagulation factors.

In the guidelines of the American College of Chest Physicians it is recommended to stop warfarin 5 days before surgery, unless the risk of bleeding is low [6]. In our experience, after routinely stopping warfarin for 5 days before surgery, we continue to observe an elevated INR on the day of surgery, in some patients. In this study we aimed at identifying easily available clinical factors that may be helpful to predict a slow normalization of the INR.

## Methods

### Study design

In this study we retrospectively reviewed the data from two sources, both consisting of patients seen by the Thrombosis Service at Hamilton Health Sciences preoperatively for bridging of anticoagulation in association with surgery or invasive procedures. The first source was a cohort of 119 patients included during 2003 to 2005 in a study on the residual activity of low-molecular-weight heparin (LMWH), used for bridging, on the day of surgery. Sixty-seven out of these fulfilled the criteria for inclusion in our study. The second source was the registry of all patients seen for perioperative bridging during the years 2005 and 2006 (n = 1,600), from which 136 consecutive eligible patients were included. Cases that were present in both sources (n = 16) were not included twice. Patients eligible for inclusion had to be anticoagulated with warfarin for at least one month before the baseline visit, which was approximately one week before the procedure or surgery. The INR at the baseline visit had to be at least 2.0. We excluded patients with no INR value obtained on the day of surgery, which corresponded to about 70% of all patients. The reason for missing INR value on the day of surgery was that the surgeon did not request it, because establishing a normalized INR was not considered crucial a) for the type of procedure or b) routinely by that surgeon. This was therefore not a selection made by us or based on patient characteristics. Another 10% of patients had to be excluded due to missing information regarding concomitant medication or other important variables. Patients who received vitamin K already at the baseline visit to insure a low INR at the time of surgery (n = 5) were excluded.

### Data capturing

We retrieved data from the registry of bridged patients at the Thrombosis Service and from the computerized medical records of the hospital as follows: patient characteristics – age, sex, body mass index (BMI) and information regarding liver disease based on previous diagnosis and/or concurrent laboratory results on serum levels of albumin, bilirubin and liver transaminases; warfarin-related data – maintenance dose of warfarin (mean, mg/week) during the month before surgery, baseline INR value, interval between last dose of warfarin and procedure/surgery, preoperative INR value, dose of vitamin K in case of high preoperative INR necessitating reversal of warfarin; concomitant medications; readily available laboratory data on hemoglobin, serum albumin, bilirubin and creatinine.

### Definitions and endpoints

The primary endpoint was "slow normalization of prothrombin time" (S) versus "expected normalization" (E). We defined S-patients as those with an INR of at least 1.6 on the day of surgery or who had INR 1.6 or higher the day before surgery and were brought back to Thrombosis Service to receive vitamin K at a dose of at least 1 mg orally to ensure a sufficiently low INR the following day.

We analyzed for the effect of individual concomitant drugs that were frequently used and had known interactions with warfarin. A typical example is amiodarone, which had a high prevalence in our population and has a well-described profound effect on the clearance of warfarin [7]. Due to the plethora of concomitant medications, we also created a "medication interaction score" in an attempt to summarize the effect of medications known to interact in a positive or negative way with warfarin. Medications known to enhance the effect of warfarin (e.g. amiodarone) received a positive score and vice versa. We classified the interacting drugs as having a moderate (1 point) or strong (2 points) effect on the pharmacodynamics of warfarin. This was based partly on the level of evidence for such an interaction according to a recent systematic overview [8] and partly on our experience of the magnitude of the interaction. Our final classification is shown in Table 1. We then added the net effects of all interacting drugs to produce the "medication interaction score".

**Table 1:** Score awarded to drugs, according to the evidence and impact of interaction with warfarin

**Enhancement, +1**	**Enhancement, +2**
acetaminophen	alcohol
acetylsalicylic acid	amiodarone
amoxicillin/clavulanate	anabolic steroids
azithromycin	cimetidine
celecoxib	ciprofloxacin
chloral hydrate	clofibrate
citalopram	cotrimoxazole
clarithromycin	entacapone
dextropropoxyphene	erythromycin
disulfiram	fenofibrate
diltiazem	fluconazole
fluorouracil	fluoxetine
fluvastatin	isoniazid
fluvoxamine	metronidazole
gemcitabine	miconazole
interferon	phenylbutazone
itraconazole	piroxicam
levamisole/fluorouracil	propafenone
levofloxacin	sulfinpyrazone
omeprazole	sulfamethoxazole-
paclitaxel	trimetoprim
phenytoin	tamoxifen
propranolol	thyroid hormone
quinidine	voriconazole
ropinirole	zileuton
sertraline	
simvastatin	
tetracycline	
tolterodine	
tramadol	

**Inhibition, -1**	**Inhibition, -2**

azathioprine	antithyroid drugs
chlordiazepoxide	barbiturates
dicloxacillin	bosentan
raloxifene	carbamazepine
ribavirin	cholestyramine
ritonavir	dichloralphenazone
sucralfate	griseofulvin
	mercaptopurine
	mesalamine
	nafcillin
	rifampin

The day of surgery is defined as Day 0, the day before surgery was Day -1 etc. Baseline INR was the value obtained 0 to 2 days before stopping warfarin. The BMI was calculated from the *body weight (kg) divided by the square of height *(*m*^2^).

### Statistical analysis

In the univariable analysis we used t-test for continuous data and the Chi square test or Fisher's exact test for binomial data. Variables with a P-value of less than 0.2 for an effect on the normalization of prothrombin time were entered into a stepwise logistic regression model. We used the SAS System, version 9.1 (SAS Institute Inc., Cary, NC, USA). A P-value of < 0.05 was considered statistically significant.

The Research Ethics Board of McMaster University and Hamilton Health Sciences approved the study as a retrospective chart review without need for obtaining informed consent from the patients.

## Results

We identified 202 consecutive patients from 2003 to 2006 that fulfilled the criteria for inclusion in the study. The characteristics of the patients are described in Table 2. There were 14 patients (7%) that fulfilled the criteria for slow normalization of INR, either due to an INR-value of at least 1.6 on the day of surgery (n = 6) or due to requirement for reversal of warfarin with vitamin K the day before surgery (n = 8). The mean INR on day -1 for those who were reversed with vitamin K was 1.95. In one case with an INR of 1.9 on the day of surgery the pacemaker implantation was postponed for one week.

**Table 2:** Characteristics of the patients according to rate of normalization of prothrombin time, and warfarin therapy

**Characteristics**	**Normal**	**Slow**	**All**	**Uni-variable p-value**	**Multi-variable p-value**
N	188	14	202		
Sex, males (%)	136(72)	11(79)	147(73)	n.s.^‡^	
Age, y*	69.1(± 10.8) *(36–89)*	73.4(± 13.2) *(48–93)*	69.4(± 11.0) *(36–93)*	0.16	0.49
BMI*	29.2(± 5.5) *(17.1–55.1)*	29.0(± 4.3) *(22.2–40.1)*	29.2(± 5.4) *(17.1–55.1)*	n.s	
Weekly warfarin dose, mg*	29.7(± 12.3) *(6.25–77)*	23.2(± 13.7) *(8.75–57.5)*	29.3(± 12.4) *(6.25–77)*	0.06	0.20
Baseline INR*	2.6(± 0.5) *(2.0–4.2)*	3.2(± 0.6) *(2.1–4.3)*	2.7(± 0.5) *(2.0–4.3)*	0.0004	0.0021
Day of last dose warfarin* (in relation to surgery)	-6.1(± 0.5) (-8 – -5)	-6.1(± 0.3) (-7 – -6)	-6.1(± 0.5)	n.s.	
Pre-operative creatinine, μmol/L*	104(± 34) (49–336)	120(± 41) (65–196)	105(± 35) (49–336)	0.09	0.40
Known liver disease (%)	7(3.7)	1(7.1)	8(4.0)	n.s.	
Medication interaction score*	1.5(± 1.3) (-1 – 5)	1.4(± 1.4) (0–4)	1.5(± 1.3) (-1 – 5)	n.s.	
Number of concomitant drugs*	7 (± 3) *(0–14)*	8 (± 3) *(4–16)*	7 (± 3) *(0–16)*	n.s.	
Amiodarone medication (%)	29(15)	2(14)	31(15)	n.s.	
Thyroid hormone treatment (%)	21(11)	2(14)	23(11)	n.s.	

The highest baseline INR-value was 4.3. Baseline INR showed a statistically significant association with slow return to normal hemostasis (p = 0.0004). There was also a statistical trend to such an association for the maintenance dose of warfarin (p = 0.06) and for serum creatinine (p = 0.09). Only four patients did not have any concomitant medications, all in the normal group. There was no effect of concomitant medications such as amiodarone or thyroid hormone medications, which were used by 15% and 11%, respectively, of our patient population and have strong enhancing interactions with warfarin. We therefore also examined the possible net effect of all concomitant medications, as assessed with the "medication interaction score" we had defined, but again no association was found.

**Figure 1:**
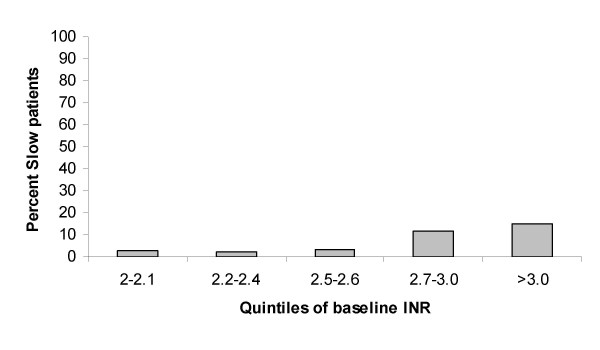
**Slow normalization with high INR**. Proportion of patients with slow normalization of prothrombin time according to baseline INR-value.

On multivariable stepwise logistic regression model, baseline INR was significantly associated with slow normalization of the INR (p = 0.0021) (Table 2). All other variables entered into the multivariable model lost significance. The proportion of patients with slow normalization of prothrombin time according to quintiles of baseline INR is shown in Fig. 1.

**Figure 2:**
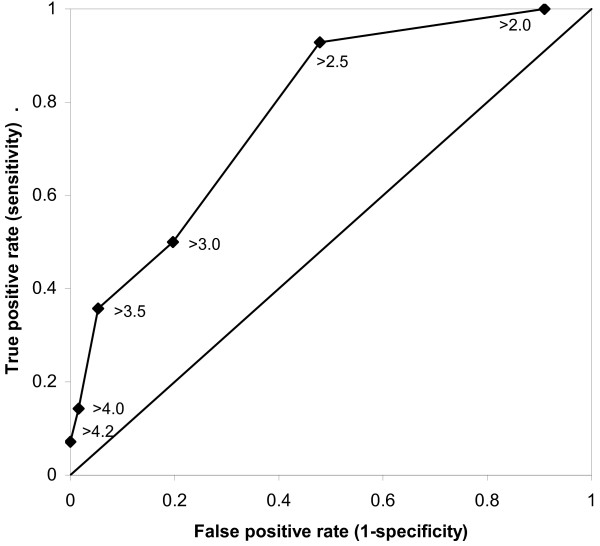
**ROC curve on predictability of baseline INR**. Receiver operating characteristics (ROC) curve for different cut-off values, as shown within the graph, of baseline INR to predict slow return to normal prothrombin time after 5 days of cessation of warfarin.

The positive predictive value (PPV) of a high baseline INR to identify slow normalization of prothrombin time was 40% for a cut-off at INR > 4.0, 33% for a cut-off at INR > 3.5, and 15% for a cut-off at INR > 3.0 but the sensitivity was low, as illustrated in the receiver operating characteristics (ROC) curve (Fig. 2).

## Discussion

In this study we have shown that 7% of the patients did not normalize their prothrombin so that the INR remained above 1.5 on the day of surgery, in spite of discontinuing their warfarin medication for 5 days. Of these cases, one patient had the surgery cancelled and 8 patients needed to return to the anticoagulant clinic for vitamin K on the day before surgery. The baseline INR value, i.e. on the day of stopping warfarin, was the only variable that was independently associated with a slow return to normal hemostasis. Age, BMI and dose of warfarin could have been expected to influence the rate of normalization but were not independent predictors.

Our results differ somewhat from those published by Hylek et al [9]. They found that in addition to the baseline ("index") INR also lower maintenance dose of warfarin, age, decompensated congestive heart failure and active cancer prolonged the normalization. Their population was, however, quite different, defined by excessive baseline INR of > 6.0 and not planned for surgery. Lubetsky et al observed that the INR at presentation in patients with excessive anticoagulation was important for the subsequent response to reversal with vitamin K [10]. Whitlock et al observed in a small study of 40 patients with warfarin discontinued 6.2 ± 1.3 days before surgery, but with half of them randomized also to receive 5 mg of vitamin K on the day of cessation, that INR decreased preoperatively to 1.2 ± 0.1 [11]. The only significant predictor for the value of individual coagulation factor levels was the time point from cessation.

The recommendation to stop warfarin for 5 days before surgery [6] is based partly on the findings in a study where INR levels were followed closely after discontinuation of warfarin [12]. Interruption of the medication for 4 doses was sufficient to reach INR of < 1.2 on the day of surgery, provided that the baseline INR was between 2.0 and 3.0, but no patient had INR above 3.0 initially.

Our patient population reflects the cardiovascular profile of our hospital, and the results may not be applicable to all patients. However, a majority of the patients treated with warfarin have a history of cardiovascular disease. In those treated with oral anticoagulants to prevent a stroke or systemic embolism after mechanical heart valve replacement or in patients with atrial fibrillation and a previous stroke, the number of days off anticoagulation before surgery should be minimized. On the other hand, many procedures require a satisfactory hemostatic capacity. It is thus of interest to find the optimal number of days that the patient should be off warfarin before such an event. The majority of our patients were bridged with therapeutic dose of the LMWH enoxaparin for the last 3 days before surgery. It has been shown that with the last dose of LMWH given a mean of 24 h before surgery, a residual effect with anti-factor Xa-levels between 0.1 and 0.86 IU/mL may still be measured on the day of surgery in 30% of patients [13]. A residual effect of the LMWH may cause a false high INR-value, but this has mainly been observed with a point-of-care method and not with standard laboratory methods [14]. In our material there was no difference in use of LMWH between patients with normal or slow normalization (data not shown).

The large proportion of males in this population may be a reflection of the cardiovascular profile of the hospital, but it is actually identical to that described in a large cohort study [15]. The average maintenance dose of warfarin was 29.3 mg/week, compared to 5.42 mg/day (37.9 mg/week) in the above-mentioned study, but the mean age of the patients was 69.4 years in our study compared to 58 years in the large cohort. The dose of warfarin is inversely associated with age [16], which may explain the lower maintenance dose in our population.

In the study by White et al [12], an association between age and the time of normalization of INR was described. In our study with about 10 times larger population, we could not confirm this association.

A retrospective study design may produce results that are flawed by selection bias. We tried to avoid this by including the majority as consecutive patients from a registry of all those seen by us before surgery. We did not have information on nutrition or alternative drugs used by the patients, so we cannot exclude such effects on the normalization of hemostasis. Another weakness is that we did not follow warfarin plasma concentration after cessation, and we are unable to exclude that some patients were non-adherent and continued to take warfarin against our instructions. The patients had, however, been evaluated carefully and educated at the preoperative visit to make sure they understood all instructions and would manage self-injections with LMWH.

Our study was not designed to evaluate the effect of the INR on the risk of bleeding during or after surgery. Patients who were not tested immediately before usually had low-risk procedures. Patients with high-risk procedures were tested and the INR corrected whenever it was too high.

The main limitation of the study is probably that we did not analyze the genetic polymorphisms of the CYP2C9 and VKOR*C1 *enzymes, due to the fact that no DNA samples were saved. The routine analysis of these polymorphisms would incur additional costs and perhaps a delay in the preoperative procedures although rapid testing is now possible. If mandatory genotyping will be widely adopted, as proposed in the US, the situation will obviously change. We were hoping to identify predictive clinical factors that could be useful in a busy clinical setting. Although the baseline INR value was significantly associated with the time to normalization of prothrombin time, it was not a sufficiently good predictor due to large overlap. A future study should therefore assess the additional predictive value of CYP2C9 and VKOR*C1 *polymorphisms.

## Conclusion

In this retrospective analysis of two cohorts with 202 patients discontinuing warfarin 6 days before surgery 14 patients (7%) had an unsatisfactory high INR prior to surgery and 8 of those required reversal with vitamin K. In multivariable analysis baseline INR, but not maintenance dose, age, BMI or concomitant medications, was an independent predictor of slow normalization of INR.

## Abbreviations

VKA: vitamin K antagonists; INR: international normalized ratio; LMWH: low molecular weight heparin; BMI: body mass index; PPV: positive predictive value; ROC: receiver operating characteristics

## Competing interests

The authors declare that they have no competing interests.

## Authors' contributions

SS designed and coordinated the study, RE and MZ retrieved the data and RE also participated in the statistical analysis, MO provided one of the cohorts, SS and RE drafted the manuscript and all authors read and approved the final manuscript.
